# EVOLVING TRENDS AND EMERGING THEMES IN GUT MICROBIOTA RESEARCH: A COMPREHENSIVE BIBLIOMETRIC ANALYSIS (2015-2024)

**DOI:** 10.1590/S0004-2803.24612025-023

**Published:** 2025-09-05

**Authors:** Naruaki OGASAWARA

**Affiliations:** 1 The Japanese Society of Internal Medicine, Editorial Department, Tokyo, Japan.

**Keywords:** Gut microbiota, dietary intervention, bibliometric analysis, Microbiota intestinal, intervenção dietética, análise bibliométrica

## Abstract

**Background:**

This study aims to analyze research trends and emerging insights into gut microbiota studies from 2015 to 2024 through bibliometric analysis techniques. By examining bibliographic data from the Web of Science (WoS) Core Collection, it seeks to identify key research topics, evolving themes, and significant shifts in gut microbiota research. The study employs co-occurrence analysis, principal component analysis (PCA), and burst detection analysis to uncover latent patterns and the development trajectory of this rapidly expanding field.

**Methods:**

This study uses a bibliometric approach to analyze 89,512 gut microbiota research articles published between 2015 and 2024 in the WoS Core Collection. Data preprocessing involved cleaning bibliographic data and identifying the 50 most frequent keywords. A co-occurrence matrix was constructed to capture keyword relationships, and a heatmap visualization illustrated these interconnections. PCA applied for dimensionality reduction, visualizing keyword distributions. Burst detection analysis using Kleinberg’s algorithm identified rapidly growing research topics. Finally, the study contextualized its findings by linking results to broader research developments and discussing future research directions and potential opportunities.

**Results:**

The bibliometric analysis of gut microbiota research from 2015 to 2024 revealed significant trends and emerging themes. The total number of publications on gut microbiota increased approximately 5.82 times during this period, indicating a rapid expansion of the field. Co-occurrence analysis identified key thematic clusters, with “diet”, “microbiome”, and “immune function” emerging as central research topics. PCA further clarified topic relationships, revealing strong associations between gut microbiota and metabolic diseases, inflammation, and neurological disorders. Burst analysis of key terms demonstrated a shift in research focus, with increasing attention on the role of gut microbiota in precision medicine, neuroinflammation, and host-microbiome interactions. These findings provide a comprehensive overview of gut microbiota research trends, offering insights into critical developments and guiding future investigations into microbiome-based therapies and disease prevention.

**Conclusion:**

This study provides a comprehensive bibliometric analysis of gut microbiota research from 2015 to 2024, highlighting key trends and emerging directions. The findings show that gut microbiota studies have expanded to include diet, health, and disease. The strong link between “diet” and “microbiota” in this study suggests dietary interventions are central to this future research. Rapidly growing keywords like “intestinal”, “disease”, and “mice” indicate a focus on translational and experimental research. These insights reveal the shifting landscape of gut microbiota research and emphasize the need for further exploration of diet-microbiota interactions, personalized nutrition, and clinical applications.

## HIGHLIGHTS

• This study analyzes data from the WoS Core Collection (2015-2024) on gut microbiota research through three analytical methods: co-occurrence analysis, principal component analysis, and burst detection analysis to uncover emerging research trends and insights.

• The findings highlight the central role of diet in gut microbiota studies, revealing frequent associations between dietary interventions and health outcomes that reflect the growing emphasis on the gut-diet-health axis. The results further indicate that nutritional interventions targeting intestinal microbiota will likely receive increasing attention in health maintenance and disease treatment domains.

• The research compares trends across different geographical regions and clarifies how cultural and dietary backgrounds influence gut microbiota research priorities. Such regional diversity promises to significantly contribute to future international collaborative efforts and cross-cultural comparative studies.

• This novel quantitative literature approach, combining three distinct analytical methods, enables a comprehensive grasp of global trends and emerging research themes in gut microbiota research, providing deeper insights unattainable through conventional analytical methods alone.

## RESUMO

**Contexto:**

Este estudo tem como objetivo analisar as tendências de pesquisa e insights emergentes em estudos sobre a microbiota intestinal de 2015 a 2024, utilizando técnicas de análise bibliométrica. Ao examinar dados bibliográficos da Web of Science (WoS) Core Collection, busca identificar tópicos de pesquisa-chave, temas em evolução e mudanças significativas na pesquisa sobre microbiota intestinal.

**Objetivo:**

O estudo emprega análise de coocorrência, análise de componentes principais (PCA) e análise de detecção de explosões para revelar padrões latentes e a trajetória de desenvolvimento desse campo em rápida expansão.

**Métodos:**

Este estudo utiliza uma abordagem bibliométrica para analisar 89.512 artigos de pesquisa sobre microbiota intestinal publicados entre 2015 e 2024 na WoS Core Collection. O pré-processamento dos dados envolveu a limpeza dos dados bibliográficos e a identificação das 50 palavras-chave mais frequentes. Uma matriz de coocorrência foi construída para capturar as relações entre as palavras-chave, e uma visualização em mapa de calor ilustrou essas interconexões. A PCA foi aplicada para redução de dimensionalidade, visualizando distribuições de palavras-chave. A análise de detecção de explosões, utilizando o algoritmo de Kleinberg, identificou tópicos de pesquisa em rápida expansão. Por fim, o estudo contextualizou suas descobertas, ligando os resultados a desenvolvimentos de pesquisa mais amplos e discutindo direções e oportunidades de pesquisa futuras.

**Resultados:**

A análise bibliométrica da pesquisa sobre microbiota intestinal de 2015 a 2024 revelou tendências significativas e temas emergentes. O número total de publicações sobre microbiota intestinal aumentou aproximadamente 5,82 vezes durante este período, indicando uma rápida expansão do campo. A análise de coocorrência identificou clusters temáticos-chave, com “dieta”, “microbioma” e “função imunológica” emergindo como tópicos centrais de pesquisa. A PCA esclareceu ainda mais as relações entre os tópicos, revelando associações fortes entre a microbiota intestinal e doenças metabólicas, inflamação e distúrbios neurológicos. A análise de explosão de termos-chave demonstrou uma mudança no foco da pesquisa, com crescente atenção ao papel da microbiota intestinal na medicina de precisão, neuroinflammation e interações hospedeiro-microbioma. Essas descobertas fornecem uma visão abrangente das tendências de pesquisa sobre microbiota intestinal, oferecendo insights sobre desenvolvimentos críticos e orientando investigações futuras sobre terapias baseadas no microbioma e prevenção de doenças.

**Conclusão:**

Este estudo fornece uma análise bibliométrica abrangente da pesquisa sobre microbiota intestinal de 2015 a 2024, destacando tendências e direções emergentes. As descobertas mostram que os estudos sobre microbiota intestinal se expandiram para incluir dieta, saúde e doenças. A forte ligação entre “dieta” e “microbiota” neste estudo sugere que intervenções dietéticas são centrais para essa pesquisa futura. Palavras-chave em rápido crescimento, como “intestinal”, “doença” e “camundongos”, indicam um foco na pesquisa translacional e experimental. Esses insights revelam a paisagem em mudança da pesquisa sobre microbiota intestinal e enfatizam a necessidade de uma exploração mais aprofundada das interações dieta-microbiota, nutrição personalizada e aplicações clínicas.

## INTRODUCTION

### Background and objectives

Recent scientific investigations highlight gut microbiota’s critical influence on human health and disease^1^ ^-^ ^3^. This complex microbial ecosystem within the gastrointestinal tract performs essential metabolic functions and modulates immune responses while responding dynamically to environmental and dietary influences. Among these, dietary patterns play a pivotal role in shaping the composition and diversity of the gut microbiota, which in turn affects metabolic and immune functions. The stability of the intestinal microflora is critical to human health. Factors such as heavy use of antibiotics, poor diet, chronic stress, and environmental pollution greatly reduce the diversity of gut microbiota, disrupting metabolic function and increasing the risk of obesity and diabetes^4^ ^-^ ^6^. A disrupted gut microbiota (dysbiosis) not only raises the risk of obesity and diabetes but also contributes to systemic inflammation, leading to immune-mediated diseases such as inflammatory bowel disease, autoimmune disorders, and cancer^7^. Additionally, abnormalities in the gut microbiota increase the risk of cardiovascular diseases^8^ ^-^ ^10^. Dietary interventions, including the consumption of specific nutrients and bioactive compounds, offer promising strategies for modulating gut microbiota and improving metabolic and immune health.

### Research challenges

Despite significant advances in understanding these microorganisms, major research challenges remain. These challenges include characterizing the full scope of microbial diversity^11^, establishing clear cause-and-effect relationships^12^ ^,^ ^13^, developing standardized research protocols^14^, defining what constitutes a healthy gut microbiota^15^ ^-^ ^17^, and establishing techniques to culture gut bacteria^18^ ^-^ ^20^. Importantly, further research is needed to elucidate how a specific diet can restore microbial diversity and functionality, thereby mitigating disease risk.

### Global research initiatives - US and Europe

Globally, studies on gut microbiota are flourishing. In Western countries, major initiatives like the Human Microbiome Project (HMP) in the United States and the MetaHIT Project in Europe have expanded scientific knowledge about the evolutionary, ecological, and metabolic significance of gut microbiota^2^ ^,^ ^21^. Metagenomic analysis techniques, particularly advanced in Western nations, enable detailed examination of gut flora diversity and functions. These analyses have revealed how distinct bacterial populations promote health or trigger disease states. Recent research demonstrates that understanding the complex mechanisms connecting gut microbiota and health establishes critical foundations for novel therapeutic approaches. The gut microbiota’s composition serves as a predictive indicator for various cardiometabolic blood markers during both fasting and postprandial states. Beyond dietary factors, lifestyle elements play a substantial role; physical exercise significantly shapes the gut microbiota’s composition^3^ ^,^ ^22^ ^,^ ^23^. Growing evidence underscores how gut microbiota imbalance (dysbiosis) influences metabolic pathways and immune function. Researchers now prioritize therapeutic strategies that target the gut microbiota. Fecal microbiota transplantation (FMT) stands out for its exceptional effectiveness against refractory Clostridioides difficile infections. The therapeutic potential of FMT extends far beyond C. difficile treatment, showing encouraging results across diverse conditions - from gastrointestinal diseases and metabolic syndromes to cancer, autoimmune disorders, infectious diseases, and various neurological and brain disorders - indicating promising research potential^24^ ^-^ ^27^.

### Regional studies in Asia

In Asia, traditional dietary habits and their impact on gut microbiota composition have been extensively studied in research on intestinal flora^28^. Japanese studies focus on fermented foods like natto and miso, along with various types of seaweed. Chinese investigations center on medicinal cuisine and traditional Chinese medicine. Korean research primarily explores fermented foods, particularly kimchi and doenjang^21^ ^,^ ^29^ ^-^ ^33^.

### Research in Latin America

In Latin America, particularly in Brazil and Mexico, research on gut microbiota is rapidly advancing. These countries are actively studying the diversity of gut flora and its impact on health, with a particular focus on how traditional diets, fermented foods (Kefir), and fruits (acai, cashews, and bananas) affect the gut microbiota^34^ ^-^ ^38^. Additionally, recent research demonstrates the profound influence of gut bacteria on lifestyle-related diseases, particularly obesity and diabetes. Several studies identify specific bacterial groups that show strong associations with obesity and diabetes, indicating that gut microbiota serves as a key mediator in the pathophysiology of these conditions. Various therapeutic approaches, including probiotics, prebiotics, and synbiotics, show promise in modulating gut microbiota and enhancing metabolic health. This emerging evidence underscores the critical role of intestinal microbiota in both the management and prevention of lifestyle-related diseases^39^ ^-^ ^41^.

### Trends and future directions

**FIGURE 1:**
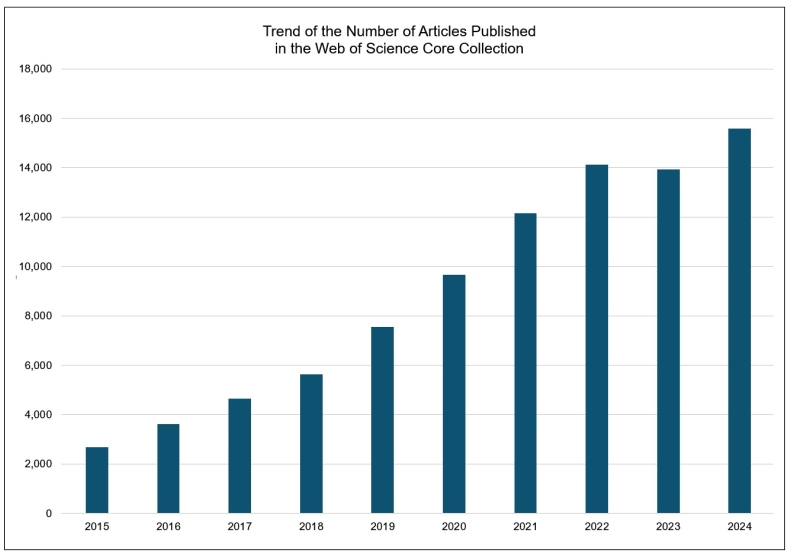
Trend of the number of articles published in the web of science core collection.

**FIGURE 2:**
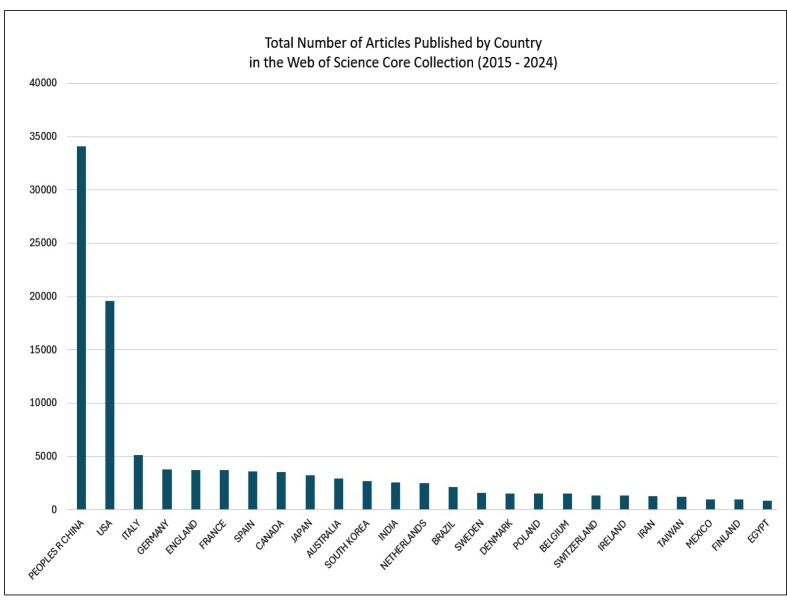
Total number of articles published by country in the web of science core collection (2015-2024).

Since 2015, the publication of gut microbiota research articles has shown a consistent upward trend (Figure 1). Analysis of WoS data for the period from 2015 to 2024 reveals that China has emerged as a dominant contributor, followed by the United States, Italy, and Germany. In Asia, Japan, South Korea, and India stand out, while Brazil leads in Latin America (Figure 2). 

To uncover trends and derive novel insights, this study employs bibliometric analysis techniques, including co-occurrence matrices, PCA, and burst detection analysis, applied to gut microbiota research from 2015 to 2024. This approach seeks to provide a comprehensive overview of the field’s evolution and identify emerging research areas.

### Scope of the Study

This study focuses on the bibliographic data of gut microbiota research articles published between 2015 and 2024, retrieved from the WoS Core Collection. It examines the most frequently occurring keywords, their interrelations, and temporal trends to visualize the dynamics of gut microbiota research and uncover latent patterns.

### Significance of the Study

By identifying key research topics in the gut microbiota domain, this study aims to bridge existing gaps in understanding and explore uncharted research areas. The diversity of approaches adopted worldwide reflects the multidimensional nature of gut microbiota research. By highlighting these differences, this study seeks to uncover novel insights, foster interdisciplinary collaboration, and propose new research methodologies.

Additionally, this work endeavors to elucidate the developmental trajectory of gut microbiota studies and provide insights that can guide future research directions. The findings are expected to stimulate innovative approaches and contribute to addressing unresolved questions in this rapidly evolving field.

## METHODS

### Data collection

#### Primary analysis

To investigate trends and insights in gut microbiota research, bibliographic data were extracted from the WoS Core Collection on January 7, 2025. The term “gut microbiota” was used as the topic search query. The data selection process included the following steps:

1. Initial search - A total of 103,453 records were retrieved using the topic search query “gut microbiota”.
2. Year filtering - The records were refined to include only those published between 2015 and 2024, resulting in 95,260 records.
3. Document type filtering - The dataset was further restricted to include only articles (original research) and review articles, yielding a final dataset of 89,512 records.

#### Secondary analysis

To perform a focused investigation on specific research topics within gut microbiota, a secondary analysis was conducted. Bibliographic data were extracted from WoS on January 14, 2025, using the topic search query “diet AND intestinal”. The data selection process included:

1. Initial search - A total of 47,340 records were retrieved using the topic search query “diet AND intestinal”.
2. Year filtering - The records were refined to include only those published between 2015 and 2024, resulting in 28,596 records.
3. Document type filtering - The dataset was further restricted to include only articles (original research) and review articles, yielding a final dataset of 28,133 records.

### Analytical environment

The extracted data are processed and analyzed using Python Programming Language (version 3.10.5) within the Integrated Development Environment (IDE) PyCharm (software version 2022.1.3).

This study used “pandas” for data manipulation and analysis, “numpy” for numerical computation, “seaborn” and “matplotlib” for data visualization, and “scikit-learn” for text feature extraction and dimension reduction. As parameters during analysis, CountVectorizer was used to exclude English stop words and extracted a maximum of 50 features in the 1-3 gram range. For dimension reduction, PCA was used with n_components set to reduce the data to two dimensions.

### Data preprocessing

To ensure data quality and relevance, the following preprocessing steps were performed:

1. Text cleaning - Titles, abstracts, and keywords were processed to remove stop words, punctuation, and other irrelevant symbols.
2. Keyword extraction - Frequently occurring keywords were identified using text mining techniques, and the top 50 most frequently occurring keywords were selected for analysis.

### Co-occurrence matrix construction

The relationships between the selected keywords were analyzed using co-occurrence matrices:

1. Matrix construction - A co-occurrence matrix was generated based on the frequency of co-occurrence of keyword pairs in the dataset.
2. Visualization - The matrix was visualized using heatmaps to highlight the strength of relationships between keywords. This approach follows methodologies described in previous studies^42^ ^-^ ^44^.

### Principal component analysis (PCA)

To reduce the dimensionality of the data and visualize keyword relationships:

1.Dimensionality reduction - PCA was applied to the co-occurrence matrix, reducing it to a two-dimensional space.
2.Visualization - The keyword distribution in the reduced space was plotted to identify clusters and patterns in research topics. This visualization approach aligns with techniques outlined in previous study^45^. In the PCA, keywords positioned positively along the principal component axes (PC1 and PC2) demonstrate substantial influence on the respective component. Moreover, keywords clustered proximally in the visualization suggest thematic interconnectedness^45^.

### Burst detection analysis

To detect emerging trends in gut microbiota research:

Temporal frequency analysis - Annual keyword frequencies were analyzed to identify significant changes over time.

Burst detection - Kleinberg’s algorithm was used to identify burst periods for the top 20 keywords^46^.

Visualization - Burst periods were plotted on a semi-logarithmic scale to emphasize trends and sudden increases in research activity.

## RESULTS

### Primary analysis

#### Co-Occurrence analysis

**FIGURE 3:**
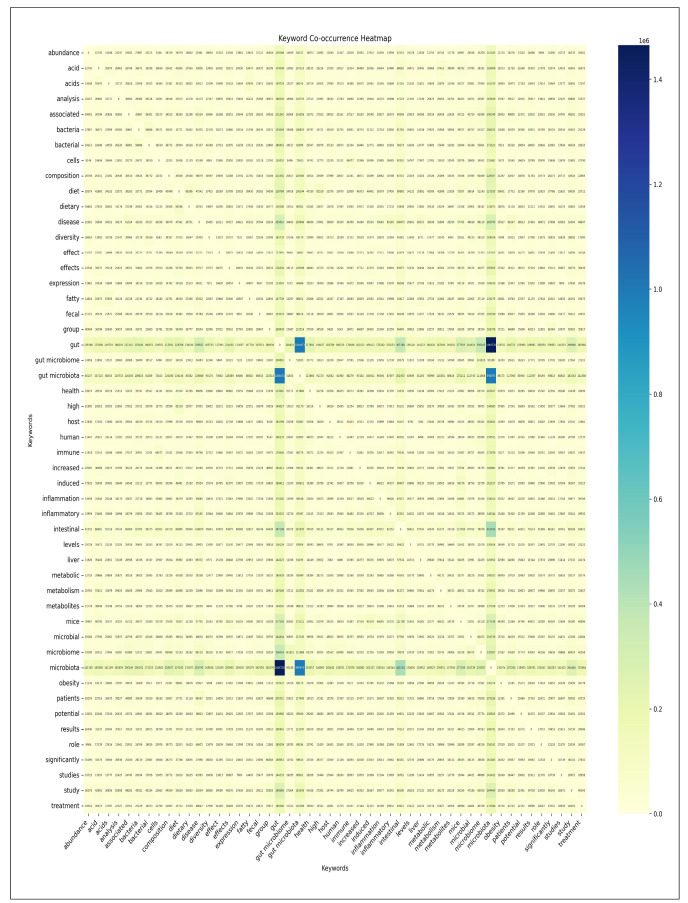
Visualization of co-occurrence analysis results - keyword co-occurrence heatmap - (primary analysis).

The analysis focused on a co-occurrence matrix derived from the top 50 most frequent keywords in bibliographic data related to gut microbiota. As anticipated, the keyword analysis revealed predictable high co-occurrence frequencies among core terms. Specifically, the most prominent co-occurrences involved: “microbiota” and “gut” (1,464,720 cases), “gut” and “gut microbiota” (1,004,471 cases), and “gut microbiota” and “microbiota” (990,975 cases) (Figure 3). As studied by Kontostathis, A. & Pottenger, W. (2002) and Becker et al. (2003), the results related to these keywords are within the expected range, as they are combinations of search keywords^47^ ^-^ ^48^.

Keyword analysis revealed significant co-occurrence patterns related to gut research. Notably, strong interrelationships between keywords were highlighted: excluding the Core Term “gut microbiota”, keywords related to “intestinal” and “microbiota” (441,534 cases), “diet” and “gut” (228,768 cases), and “diet” and “microbiota” (227,185 cases) (Figure 3). These co-occurrence patterns indicate that diet, gut, and microbiota are recent research topics of interest.

#### Principal component analysis (PCA)

**FIGURE 4:**
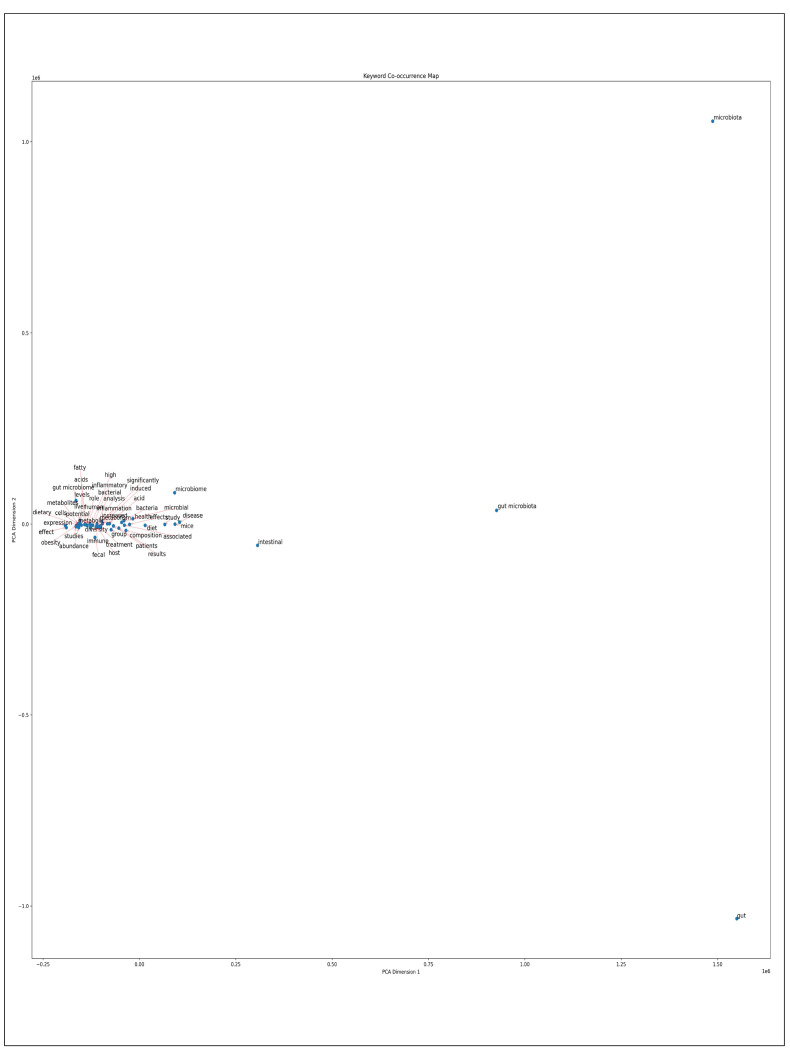
Principal component analysis (primary analysis).

The study analyzed keyword distributions using PCA based on a co-occurrence matrix. The first principal component axis (PC1) featured the core term “gut microbiota”, capturing the primary research focus. The second principal component axis (PC2) revealed interconnected themes, including “diet”, “health”, and “immune”, highlighting the multifaceted nature of gut microbiota research (Figure 4).

### Burst detection analysis

#### Identification of rapidly growing research topics through burst analysis

**FIGURE 5:**
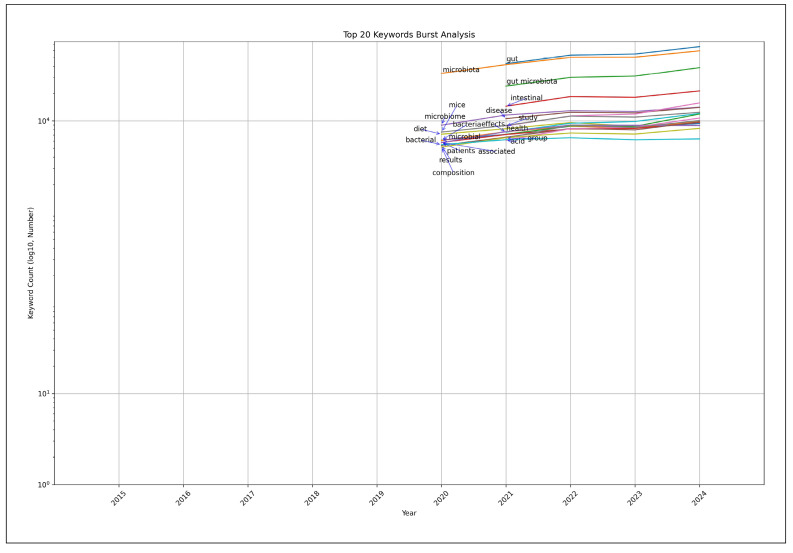
Top 20 keywords burst analysis (primary analysis results visualization).

Burst detection analysis using Kleinberg’s algorithm^46^ for gut microbiota research from 2015 to 2024 revealed a sustained surge in “gut microbiota” research throughout the period, with a notable acceleration after 2018 (Table 1). This surge aligns with the global increase in gut microbiota research publications (Figure 1). Further analysis identified several keywords that gained significant research momentum (Table 1, FIGURE 5).

**TABLE 1:** Top 50 burst detection analysis results (primary analysis).

Ano	2015	2016	2017	2018	2019	2020	2021	2022	2023	2024
abundance	466	762	1013	1546	2454	3344	4431	5891	5985	7222
acid	1021	1385	1969	2500	3253	4798	6104	8218	8517	10749
acids	775	1120	1708	2145	2917	4007	5032	6512	6105	7185
analysis	912	1138	1624	2099	2783	3793	5137	6807	7603	10699
associated	1571	2211	2743	3405	4551	5795	7083	8158	8254	9626
bacteria	2090	2711	3437	3811	4792	6146	7113	8735	8495	9346
bacterial	2102	2521	3024	3779	4396	5477	6211	6524	6197	6337
cells	1588	2177	2902	3205	3651	4466	5409	6110	6516	7030
composition	1302	1711	2369	3112	4230	5152	6544	7358	7165	8271
diet	1736	2528	3273	3937	5602	7137	8203	9585	8717	9659
dietary	955	1346	1755	2252	3110	4236	5095	6065	5898	6969
disease	2574	3560	4716	5371	6492	8142	10587	12444	12260	14047
diversity	1046	1451	1810	2482	3217	4183	5139	6060	5663	6406
effect	670	1076	1353	1813	2365	3439	4486	5583	5758	6091
effects	1294	1693	2409	2999	4354	6047	7514	9418	9844	12075
expression	909	1224	1687	2071	2606	3711	4283	5426	5427	6492
fatty	824	1157	1690	2137	3069	4055	5239	6416	6219	7085
fecal	1153	1563	1904	2576	3191	4334	5338	6264	6020	6621
group	842	1215	1635	2260	3503	5021	6458	8955	8466	10102
gut	7544	10383	13779	17744	24465	32671	42924	53093	54675	65961
gut microbiome	606	891	1265	1710	2665	3486	4815	5623	5629	6546
gut microbiota	3919	5613	7502	9738	13773	18400	24085	30074	31126	38763
health	1405	1844	2566	2995	4178	5779	7114	8792	8709	11883
high	1014	1481	1969	2517	3590	4699	5202	6689	6353	7175
host	1760	2369	2869	3196	3872	4680	5528	6085	5776	6015
human	2200	2285	3211	3280	4167	4872	5831	6074	5679	6358
immune	1593	2006	2639	2949	3806	4668	6007	6866	6970	8435
increased	1119	1491	2038	2562	3569	4739	5871	7678	7343	8095
induced	865	1270	1791	2294	3290	4370	5768	7429	7246	8821
inflammation	1200	1560	2233	2713	3557	4591	5942	7175	7269	8693
inflammatory	1141	1640	2130	2604	3119	4153	5576	6891	7166	9089
intestinal	3346	4439	5456	6860	8869	11123	14497	18504	18160	21341
levels	805	1179	1471	1956	2753	3772	4598	6093	6292	8661
liver	751	1168	1410	1637	2493	3370	4902	6045	6270	7638
metabolic	1281	1658	2153	2488	3093	4156	5038	6176	5752	7409
metabolism	1012	1287	1787	2464	3232	4393	5514	8054	7736	9937
metabolites	431	696	1070	1271	1948	2803	3971	5972	6264	8232
mice	1565	2413	3320	4086	5337	7535	8902	11273	11005	12433
microbial	1791	2415	3147	3872	4765	6095	7602	8731	8387	9824
microbiome	1974	2730	3868	5056	7148	8970	11597	12932	12698	14148
microbiota	8611	11989	15377	19498	25578	33449	41802	50279	50392	59336
obesity	1317	1690	2078	2315	3033	3989	4269	5518	4975	5450
patients	1167	1832	2274	3145	4414	5764	7612	8972	8934	8892
potential	724	1017	1362	1777	2483	3461	4630	5902	6740	10322
results	1108	1602	2130	2747	3679	5379	6611	8242	7964	9771
role	1091	1568	1885	2308	3021	3852	5128	5844	6165	7458
significantly	786	1113	1489	2148	3196	4242	5646	7434	7244	8645
studies	1058	1408	1917	2229	3016	3924	5081	5827	5927	6486
study	1422	1983	2647	3592	4977	6806	8800	11340	11888	15761
treatment	989	1465	1882	2503	3438	4654	6164	7714	7819	9252

After excluding core terms such as “microbiota” and “gut microbiota”, the following research keywords emerged as focal points (Figure 5):

-Intestinal (2021-2024)
- Disease (2021-2024)
- Mice (2020-2024)
- Diet (2020-2024)
- Effects (2021-2024)
- Health (2021-2024)

These emerging keywords reflect the shifting research priorities in intestinal microbiota studies over recent years. The burst detection analysis results, when plotted on a semi-logarithmic scale, clearly illustrate the evolutionary trajectory of novel research directions in gut microbiota investigations.

#### Summary of primary analysis

The core terms “gut microbiota” and “microbiota” represent central research keywords, while topics such as health, disease, diet, and immunity have emerged as critical directions in gut microbiota research. The keyword “diet” appeared consistently across all primary analyses (co-occurrence analysis, PCA analysis, and burst detection analysis), warranting a secondary analysis to examine its relationships with other research topics. This deeper investigation aims to uncover potential new technologies and underlying themes connected to dietary research in the gut microbiota field.

### Secondary analysis

#### Co-Occurrence analysis

**FIGURE 6:**
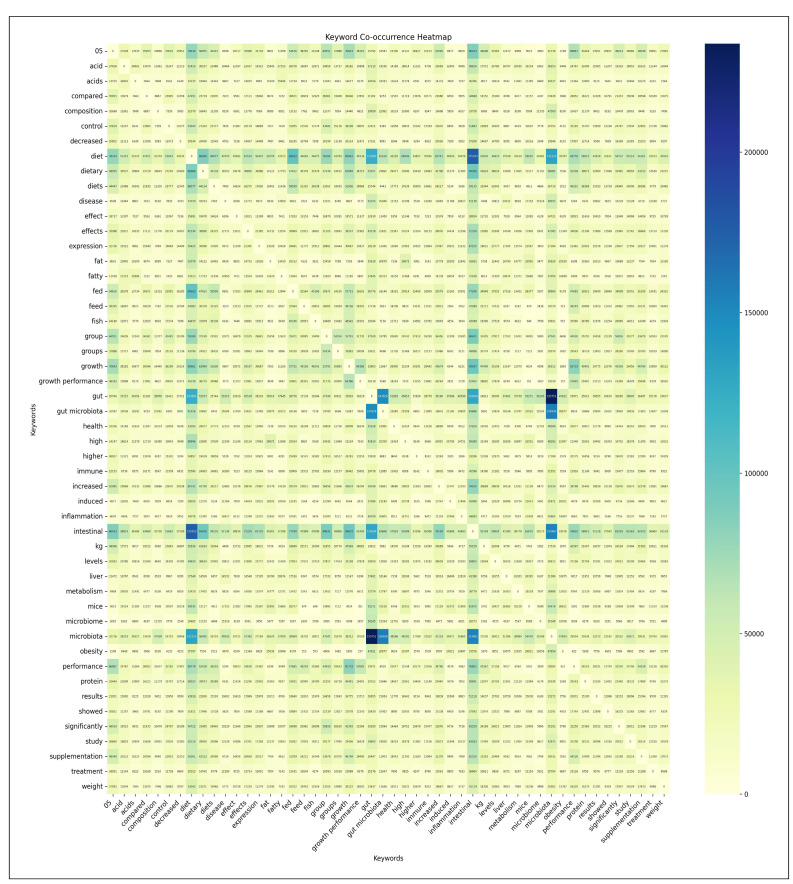
Visualization of co-occurrence analysis results - keyword co-occurrence heatmap - (secondary analysis).

A topic search using the keywords “diet AND intestinal” in the WoS Core Collection facilitated the creation of a co-occurrence matrix from the bibliographic data. The analysis revealed significant patterns in keyword associations. The strongest co-occurrence emerged between “microbiota” and “gut” (233,751 cases), while “microbiota” and “gut microbiota” ranked third (156,508 cases). This pattern indicates robust connections between dietary research and gastrointestinal microbiological studies (Figure 6).

The keyword co-occurrence analysis highlighted several significant relationships:

The association between “diet” and “fed” (99,922 cases) reflects extensive animal model studies examining dietary impacts on intestinal bacteria. The relationship between “growth” and “intestinal” (95,637 cases) indicates substantial research into bacterial influences on human and cellular development. The “intestinal” and “dietary” connection (94,930 cases) demonstrates active investigation into how dietary components affect bacterial diversity and function within the intestinal environment (Figure 6).

Further significant correlations emerged between “effects” and “intestinal” (71,320 cases), suggesting a detailed examination of bacterial metabolites’ impact on host metabolism and immune responses. The link between “supplementation” and “intestinal” (62,520 cases) points to extensive research on probiotic and prebiotic interventions. The co-occurrence of “health” and “intestinal” (57,929 cases) underscores ongoing investigation into the broader implications of intestinal bacteria for human health (Figure 6).

This analysis highlights the field’s focus on understanding the complex interactions between diet, intestinal microbiota, and human health, with particular emphasis on intervention strategies and health outcomes.

#### Principal component analysis (PCA)

**FIGURE 7:**
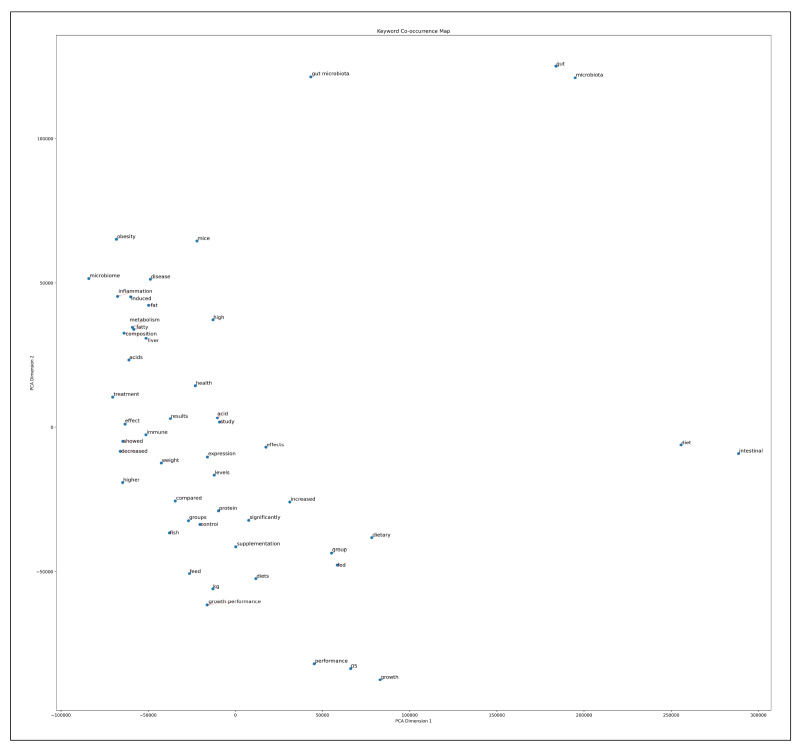
Principal component analysis (secondary analysis).

The PCA, based on the co-occurrence matrix, revealed distinct keyword distributions in two-dimensional space. The first principal component axis (PC1) showed strong correlations with intestinal microbiota-related keywords, including “gut microbiota,” “gut,” and “microbiota”. This primary analysis identified Core Terms such as “diet” and “intestinal” as central themes in intestinal microbiota research. Along the second principal component axis (PC2), health and pathological keywords clustered in the positive direction, including “obesity”, “microbiome”, “disease”, “inflammation”, and “fat”. Conversely, the negative direction of PC2 contained keywords associated with dietary interventions and growth metrics, such as “protein”, “control”, “fish”, “dietary”, “supplementation”, “diets”, “group”, and “growth performance” (Figure 7).

### Burst detection analysis

#### Identification of rapidly growing research topics through burst analysis

**FIGURE 8:**
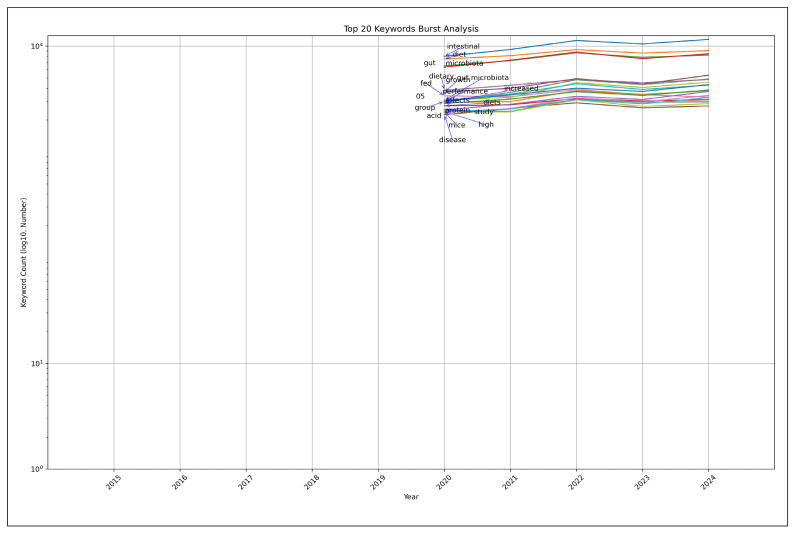
Top 20 keywords burst analysis (secondary analysis results visualization).

Burst detection analysis using Kleinberg’s algorithm^46^ revealed a consistent annual increase in gut microbiota research from 2015 to 2024, indicating significant growth in this field (Table 2). Basic terms such as “intestinal”, “microbiota”, and “gut” showed particularly notable increases in frequency, reflecting heightened research interest. Further analysis identified specific research keywords that gained prominence during this period (Table 2, Figure 8). Beyond the fundamental terms and Core Term bursts like “diet” and “intestinal”, several key research keywords emerged with sustained prominence (Figure 8):

-Growth (2020-2024)
- Fed (2020-2024)
- Performance (2020-2024)
- Increased (2020-2024)
- Effects (2020-2024)
- Protein (2020-2024)
- Mice (2020-2024)
- Disease (2020-2024)

**TABLE 2:** Top 50 burst detection analysis results (secondary analysis).

Ano	2015	2016	2017	2018	2019	2020	2021	2022	2023	2024
05	1016	1272	1465	1773	2328	3095	3281	4514	4076	4565
acid	974	1170	1297	1483	1794	2351	2577	3252	2970	3432
acids	540	705	895	1010	1321	1624	1785	2127	1994	1967
compared	734	829	962	1104	1401	1860	1924	2402	2063	2365
composition	549	633	760	898	1231	1496	1590	1917	1733	1786
control	885	1049	1107	1240	1730	2224	2283	2682	2316	2595
decreased	526	593	758	779	1026	1438	1472	1829	1611	1730
diet	3282	3775	4262	4872	6228	7542	8117	9255	8584	9052
dietary	1578	1810	2168	2338	3133	3828	4272	4841	4514	4836
diets	1130	1352	1556	1680	2125	2734	2798	3201	3041	3124
disease	1406	1393	1673	1695	2044	2247	2583	2923	2619	2726
effect	685	861	886	1091	1289	1607	1888	2044	1892	1885
effects	1121	1226	1466	1654	2198	2881	3161	3723	3432	3850
expression	981	1072	1222	1413	1812	2337	2418	3027	2617	3135
fat	735	850	875	1089	1508	1656	1926	2209	1989	2248
fatty	585	766	865	970	1347	1642	1837	2180	1999	2062
fed	1496	1802	1854	2201	2694	3400	3330	3895	3491	3304
feed	747	829	996	1128	1527	2009	2458	2583	2588	2782
fish	609	825	763	978	1303	1859	2067	2364	2209	2090
group	873	1094	1228	1507	2011	2999	3654	4820	4334	4895
groups	587	758	819	967	1385	1763	2139	2627	2520	3029
growth	1094	1364	1613	1886	2699	3675	4008	4930	4387	5314
growth performance	505	618	851	995	1447	1951	2203	2710	2372	2785
gut	1897	2472	3016	3645	4930	6322	7352	8778	7613	8451
gut microbiota	781	1233	1357	1792	2509	3084	3520	4411	3904	4289
health	592	739	904	1014	1541	2075	2562	3079	2964	3747
high	929	1075	1164	1468	1905	2404	2550	3141	2861	3261
higher	555	666	780	899	1130	1472	1490	1820	1737	1681
immune	689	785	975	1020	1352	1689	2029	2327	2212	2157
increased	1219	1310	1590	1731	2243	2949	2997	3800	3506	3794
induced	737	827	1013	1065	1382	1596	1842	2252	1988	2088
inflammation	564	731	772	953	1300	1573	1858	2101	1890	2127
intestinal	3494	3858	4622	5023	6701	8002	9288	11289	10479	11554
kg	728	849	999	1047	1596	2199	2419	2923	2482	2939
levels	1001	1087	1251	1455	1634	2161	2314	2885	2761	3656
liver	607	789	865	860	1268	1545	1946	2374	2409	2727
metabolism	546	692	759	917	1126	1569	1664	2407	2165	2621
mice	1083	1358	1424	1644	2007	2395	2397	3119	2701	2867
microbiome	495	590	874	838	1225	1569	1903	2048	1770	1793
microbiota	1951	2920	3256	4039	5339	6500	7287	8642	7835	8229
obesity	736	921	1032	1059	1302	1511	1674	1943	1765	1971
performance	944	1018	1456	1558	2212	3021	3470	4009	3737	4334
protein	1027	1294	1473	1545	1910	2507	2419	3161	2961	2984
results	760	919	1071	1207	1571	2034	2194	2700	2391	2692
showed	422	565	626	767	979	1384	1586	2137	1947	2062
significantly	760	874	1054	1232	1681	2249	2417	3499	3115	3521
study	923	1089	1294	1465	1969	2504	2831	3360	3134	3741
supplementation	777	877	1326	1255	1763	2536	2589	3161	2828	3208
treatment	677	737	906	904	1264	1579	1733	2066	1820	1961
weight	758	882	1026	1092	1430	1883	1890	2192	2185	2432

These findings point to a distinct shift in research focus toward understanding the mechanisms through which diet-microbiota interactions influence health and disease processes. This trend reflects the growing recognition of gut microbiota as a central factor in human health and disease prevention.

#### Summary of secondary analysis

A secondary analysis explored the relationship between “diet” and “intestinal” - a key research keyword identified in the primary analysis - and other research topics. The co-occurrence analysis revealed strong associations between “microbiota” and “gut” (233,751 cases) and between “microbiota” and “gut microbiota” (156,508 cases), highlighting the significant connection between dietary and gut microbiota research. The PCA demonstrated that gut microbiota-related keywords, including “gut microbiota”, “gut”, and “microbiota”, exhibited strong correlations along principal component axis 1 (PC1), while health and dietary intervention-related keywords clustered along principal component axis 2 (PC2). The burst detection analysis identified robust connections between gut microbiota keywords and health-related terms, indicating active research into health effects and disease prevention through diet-microbiota interactions. These findings suggest that dietary interventions and nutritional approaches may offer critical insights into diet’s impact on health and disease.

## DISCUSSION

### Key findings and implications

This bibliometric analysis of gut microbiota research from 2015 to 2024 identifies significant trends and emerging themes. By integrating co-occurrence matrices, PCA, and burst detection analysis, this study provides a comprehensive view of the evolving research landscape. The combination of these three analytical techniques represents an innovative bibliometric approach, which has been successfully applied in various scientific fields^49^ ^-^ ^51^. This study applies this method to gut microbiota research, offering new insights into its development over the past decade.

Research on gut microbiota is being conducted all over the world (Figure 2) and has attracted much attention in the field of gastroenterology^52^ ^-^ ^54^. The number of gut microbiota research articles has increased approximately 5.82 times from 2015 to 2024 (Figure 1). Among them, “diet” has been an inseparable trend in the study of gut microbiota in the last decade^55^ ^-^ ^58^. In this study, diet also emerged as an important research keyword in the primary and secondary analyses, highlighting its impact on gut microbiota research. The growing emphasis on dietary interventions reflects advancements in nutritional science, particularly regarding probiotics, prebiotics, and dietary fiber^35^ ^,^ ^40^ ^,^ ^59^ ^-^ ^61^. The increasing recognition of diet as a modifiable factor influencing gut microbiota diversity underscores its importance in disease prevention and therapeutic strategies^60^.

Co-occurrence analysis and PCA revealed thematic clustering of research topics, with diet-related keywords frequently appearing alongside metabolic diseases, immune function, and microbiome modulation. This pattern aligns with the broader scientific consensus that dietary interventions play a critical role in shaping gut microbiota and subsequently influencing health outcomes. The burst detection analysis identified several prominent research keywords, including “intestinal”, “disease”, “mice”, “diet”, “effects”, and “health”, indicating a significant shift toward investigating gut microbiota’s role in health and disease in the primary analysis. The secondary burst analysis uncovered additional key terms with strong burst signals, such as “growth”, “fed”, “performance”, “increased”, “effects”, “protein”, and “mice”. This pattern demonstrates heightened interest in understanding how diet-microbiota interactions affect physiological outcomes. The frequent appearance of terms like “diet” and “intestinal” points to increased attention on dietary approaches for gut microbiota modification, potentially advancing both disease prevention and health enhancement strategies.

These burst trends underscore the critical role of gut microbiota in human health, particularly through mechanistic studies exploring diet-induced microbial changes. The study’s findings support the development of personalized nutrition and probiotic interventions that aim to optimize gut microbiota composition and enhance health outcomes. This research provides valuable insights for clinicians and companies to advance therapeutic strategies and product development in gut health. Further research should examine these complex interactions to establish precise nutritional interventions that maximize gut microbiota-mediated health benefits.

### The study period (2015-2024)

The study period spanning 2015 to 2024 captures the most recent advancements in gut microbiota research. The field has experienced exponential growth since the early 2010s, driven by major initiatives such as the HMP and advances in high-throughput sequencing technologies^62^ ^-^ ^64^. This timeframe effectively captures the transition from exploratory microbiome research to translational applications, including precision nutrition, microbiome-based therapies, and personalized medicine^65^ ^-^ ^67^. The increasing prevalence of microbiota-targeted interventions and their integration into clinical practice further justifies this temporal scope. The period also corresponds with the rapid expansion of global research efforts, particularly in Asia, Europe, and Latin America, reflecting the internationalization of gut microbiota studies.

### Originality and strengths of this study

#### Novel methodological approach - bibliometric analysis with advanced techniques

This research introduces a novel approach to gut microbiota studies by employing bibliometric analysis to systematically track research evolution from 2015 to 2024, departing from conventional experimental or clinical investigations. The methodology incorporates co-occurrence matrices to identify relationships among key research topics, PCA to classify emerging themes, and burst detection to pinpoint rapidly growing research areas. This approach provides one of the first comprehensive, quantitative assessments of global research patterns in gut microbiota. The methodological innovation distinguishes this work from prior studies, offering a structured, data-driven understanding of the field’s development.

#### Comparative global perspective - integrating regional studies

While previous studies have typically focused on gut microbiota research within specific countries or regions, this investigation integrates research data from North America, Europe, Asia, and Latin America. This comprehensive approach enables comparative analysis of dominant research themes across different regions, yields insights into how dietary patterns in various cultures influence research priorities and creates a global mapping of scientific collaborations that illuminates cross-regional interactions. The worldwide perspective offers a holistic view of gut microbiota research evolution across diverse geopolitical and cultural contexts.

#### Identifying underexplored research areas and future directions

This investigation advances beyond a mere synthesis of previous research by identifying emerging fields and critical knowledge gaps in gut microbiota studies. The scientific literature demonstrates a shift from traditional topics like probiotics and fermented foods toward innovative areas such as postbiotics, next-generation probiotics, and gut virome interactions. 

The temporal analysis presented here provides strategic direction for policymakers, funding agencies, and researchers to identify promising research opportunities. Based on previous research about leading researchers in gastroenterology who have been studied earlier and the significance of promoting research collaboration^68^. This forward-looking perspective not only documents the historical development of gut microbiota research but also helps shape its future trajectory.

#### Contribution to research methodology in gut microbiota studies

Bibliometric analysis has found wide application across scientific fields, yet researchers have not fully explored its potential in gut microbiota studies. This investigation demonstrates how quantitative analytical techniques can effectively map knowledge structures and introduces a complementary research paradigm to traditional microbiome approaches. The systematic and objective methodology for tracking knowledge evolution serves as a model for scientometric analysis in microbiological research. By bri­dging the disciplines of microbiology and bibliometrics, this study establishes a novel methodological framework that future researchers can adapt for investigations in gut microbiota and related fields.

#### Future directions

Future research should expand the dataset to include other major databases such as Scopus and PubMed could help mitigate selection bias and provide a more comprehensive view of global research trends. Further exploration of regional research variations, particularly in developing nations, could offer valuable insights into the cultural and dietary factors influencing gut microbiota research trajectories. The integration of artificial intelligence and machine learning techniques in bibliometric analysis could enhance the predictive capacity of research trend identification, allowing for more refined forecasting of emerging topics in gut microbiota studies.

#### Key findings from discussion

This study provides a structured overview of gut microbiota research from 2015 to 2024, highlighting the centrality of diet in shaping research themes and identifying key trends through bibliometric analysis. The study period effectively captures recent advancements, though database constraints must be considered when interpreting the results. Future studies incorporating multi-omics approaches and broader data sources will further refine the understanding of gut microbiota research and its evolving impact on human health.

#### Limitations

Several limitations warrant acknowledgment in this study. First, reliance on the WoS Core Collection may introduce selection bias. While WoS serves as a reputable and widely used database for bibliometric studies, it does not encompass all relevant research outputs, particularly those published in regional or non-indexed journals. This limitation may lead to the underrepresentation of studies from certain geographical regions or emerging research communities.

Second, while the keyword-based co-occurrence method effectively identifies major research themes, it cannot capture the full complexity of gut microbiota interactions. Many microbiome studies employ metagenomic, metabolomic, and multi-omics approaches, which may not be fully represented through keyword analysis alone. Additionally, the temporal nature of burst detection analysis, while useful for identifying emerging trends, does not account for underlying methodological advancements or shifts in research paradigms that may influence keyword frequencies.

## CONCLUSION

This bibliometric analysis of gut microbiota research from 2015 to 2024 provides a comprehensive overview of the evolving trends and emerging focal points within the field. Through the application of co-occurrence analysis, PCA and burst detection analysis, this study identified key thematic structures and research trajectories that shape contemporary gut microbiota studies.

The co-occurrence analysis revealed the foundational role of “gut microbiota” and “microbiota” as core research terms, with strong associations emerging between “diet”, “intestinal”, and “health.” These interconnections underscore the centrality of diet in shaping gut microbiota composition and function, aligning with a growing emphasis on the gut-diet-health axis in microbiota research. The PCA further substantiated this relationship by demonstrating a distinct clustering of dietary and health-related keywords, positioning “diet” as a pivotal factor influencing intestinal microbiota dynamics.

The burst detection analysis identified research topics that have experienced significant growth in recent years, with key terms such as “intestinal”, “disease”, “mice”, “diet”, “effects”, and “health” showing notable increases in frequency. These findings indicate a shift towards translational research, with a heightened focus on understanding the implications of gut microbiota on human health and disease. The rapid emergence of these topics suggests an increasing recognition of gut microbiota as a critical determinant in various physiological and pathological conditions, paving the way for novel therapeutic strategies and dietary interventions.

A secondary analysis focusing on the relationship between diet and intestinal microbiota reinforced the prominence of dietary research within the field. Strong co-occurrence patterns between “diet” and terms such as “intestinal”, “effects”, and “supplementation” reflect the growing interest in dietary modulation of microbiota and its potential health benefits. PCA results further highlighted the divergence between microbiota-related disease research and dietary intervention studies, suggesting that future investigations may increasingly explore targeted nutritional approaches for microbiota modulation.

This study provides valuable insights into the key themes and emerging directions in gut microbiota research over the past decade. The integration of bibliometric methods, including co-occurrence analysis, PCA, and burst detection analysis, has enabled a structured exploration of research trends, shedding light on the evolving landscape of this field.

As gut microbiota research continues to expand, future studies may benefit from a more exploration of microbiota-host interactions, the role of specific dietary components, and the translation of microbiota-based interventions into clinical applications. Additionally, these findings highlight the shifting landscape of gut microbiota research and underscore the promising future of personalized nutrition, dietary interventions, and clinical applications. By identifying current research priorities and gaps, this analysis contributes to a deeper understanding of the field and its potential trajectory in the coming years.

In 2015, the “Precision Medicine Initiative” was announced by U.S. President Barack Obama in his State of the Union address, attracting worldwide attention. Over the past decade, advances in microbiome research and nutritional science have paved the way for the emergence of the Precision Nutrition and Precision Diet, where dietary interventions are tailored to an individual’s genetic and microbial profile. As this field evolves, an interdisciplinary approach becomes essential, with the intestinal microflora as a central axis. This requires collaboration not only among physicians (gastroenterologists) but also nutritionists, chefs, nurses, and data analysts to develop evidence-based, personalized dietary strategies.
